# Vaginal Intraepithelial Neoplasia (VaIN)—A Retrospective Cohort Analysis of Epidemiology, Risk Factors, and Management in an Academic Clinical Center

**DOI:** 10.3390/jcm14155386

**Published:** 2025-07-30

**Authors:** Barbara Suchońska, Franciszek Ługowski, Magdalena Papież, Artur Ludwin

**Affiliations:** 1st Department of Obstetrics and Gynecology, Medical University of Warsaw, 02-015 Warsaw, Poland

**Keywords:** vaginal intraepithelial neoplasia, vaginal cancer, human papillomavirus, cervical neoplasia

## Abstract

**Background**: Vaginal intraepithelial neoplasia (VaIN) is a rare but potentially precancerous condition strongly associated with human papillomavirus (HPV) infection. Despite increased detection rates due to HPV screening and colposcopy, diagnosis and management remain challenging. This study aimed to evaluate the epidemiological characteristics, risk factors, and outcomes of VaIN in patients referred to a tertiary academic center. **Methods**: We conducted a retrospective analysis of 48 patients who underwent colposcopy-directed vaginal biopsies between January 2019 and June 2024 at the Medical University of Warsaw. Data collected included patient demographics, HPV status, cytology, histopathology, and treatment outcomes. Patients were grouped based on the presence and grade of VaIN (VaIN 1 vs. VaIN 2/3). Statistical analyses were performed using SPSS software. **Results**: VaIN was diagnosed in 24 patients (50%), VaIN was confirmed in half of the cohort, VaIN 2 in 30%, and VaIN 3 in 18% of cases. HPV infection and prior cervical pathology were significantly associated with VaIN diagnosis (P = 0.03 and P = 0.05, respectively), and high-risk HPV infection correlated with higher-grade lesions (P = 0.04). Among VaIN 2+ cases, most patients required laser ablation or surgical excision, while VaIN 1 often regressed spontaneously. Regression occurred in 11 cases, and high-risk HPV infection was inversely associated with spontaneous regression (P = 0.04). **Conclusions**: This study confirms the central role of HPV, particularly high-risk subtypes, in VaIN pathogenesis. Conservative management may be appropriate for VaIN 1, while VaIN 2+ requires active intervention. HPV genotyping should be integrated into diagnostic workups, and long-term follow-up is essential due to the risks of persistence and recurrence.

## 1. Introduction

Vaginal intraepithelial neoplasia (VaIN) is a rare entity associated with human papillomavirus (HPV) infection [1]. It was first described by Graham and Meigs in 1952 [2]. Its prevalence is estimated at approximately 2–3 per 10,000 women and accounts for approximately 0.4% of precancerous lesions of the lower female genital tract [3]. According to Sillman et al., the incidence of VaIN is 100 times lower than that of cervical intraepithelial neoplasia [4]. Moreover, VaIN lesions can lead to vaginal cancer [5]. Importantly, vaginal cancer is the rarest malignancy of the anogenital region, making its precancerous lesions even less common than those of the cervix or vulva [6]. VaIN usually has no noticeable symptoms and is often diagnosed incidentally based on abnormal cytology or histopathology results [6]. Currently, there are no screening tests for vaginal cancer and precancerous lesions [7]. Therefore, they are often difficult to detect and are commonly missed by physicians. The increased availability of cytology, colposcopy, and HPV screening has resulted in a higher detection rate than before [8,9]. Several important risk factors for VaIN have been identified, such as HPV infection, increased diversity of vaginal microbiota, oral contraception, immunodeficiency, smoking, or *Chlamydia trachomatis* infection [10,11,12,13]. Moreover, a past hysterectomy is also associated with the occurrence of VaIN. In those cases, VaIN is often multifocal, present in the parietal horns or the vaginal stump, which makes it difficult to detect [14,15]. In addition, the cervixes of these women are usually small with small cervical canals, which makes it difficult to perform loop electrosurgical excision (LEEP) [13]. The highest incidence of infection occurs in 20-year-old women, but approximately 80% of sexually active women have been infected by the age of 50 [16].

**Figure 1:**
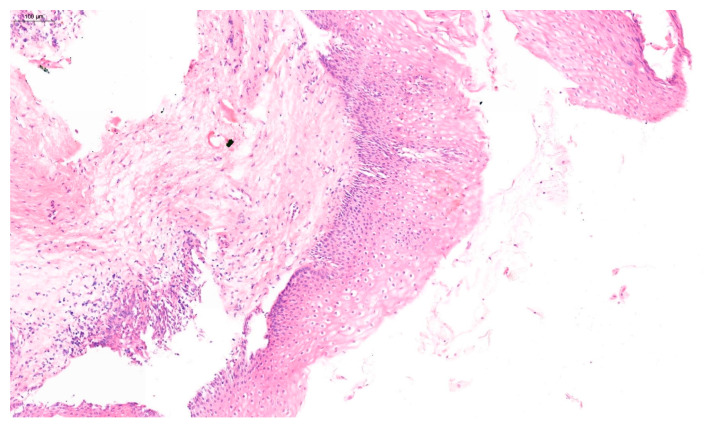
Histopathological image of VaIN 1, showing mild nuclear atypia confined to the lower third of the vaginal squamous epithelium, without evidence of invasion. Hematoxylin and eosin stain (H&E), original magnification ×200.

**Figure 2:**
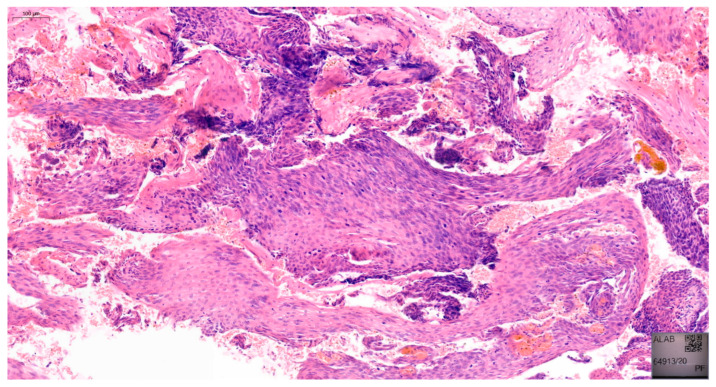
Histopathological image of VaIN 2/3, demonstrating marked nuclear pleomorphism and loss of maturation involving more than two-thirds of the epithelial thickness. Hematoxylin and eosin stain (H&E), original magnification ×200.

Furthermore, in approximately 75% of women, VaIN coexists with cervical or vulvar cancer [17]. The 2020 World Health Organization (WHO) classification distinguishes between low-grade squamous intraepithelial lesions (LSIL), which include VaIN 1, and high-grade squamous intraepithelial lesions (HSIL), including VaIN 2 and VaIN 3 [18]. Examples of VaIN 1 and VaIN 2+ lesions in patients from our cohort are shown in Figure 1 and Figure 2, respectively. Numerous studies have reported the risk of HSIL progressing to vaginal cancer ranging from 2% to 12% [19,20]. VaIN is treated in several ways, including CO_2_ laser ablation, photodynamic therapy, topical imiquimod, excisional surgery, electrocoagulation diathermy, or brachytherapy [9,21,22]. Therefore, VaIN is still a major diagnostic and therapeutic challenge, and further research is needed.

This study aimed to evaluate the epidemiological characteristics, risk factors, and outcomes of VaIN in patients referred to a tertiary academic center.

## 2. Materials and Methods

### 2.1. Study Design and Setting

The study was retrospective and included patients with an abnormal result of colposcopy for vaginal wall evaluation between 1 January 2019 and 30 June 2024 at the 1st Department of Obstetrics and Gynaecology, Warsaw Medical University. Patients were not excluded based on age, medical history, current hormonal profile, or other factors. The study protocol was reviewed and approved by the Ethics and Research Committee of the Medical University of Warsaw (approval number AKBE/6/2020). The medical records of 48 women diagnosed with VaIN 1, 2, and 3 during the mentioned period were analyzed to obtain the following data: age, number of pregnancies, current and previous cytology results, history of hysterectomy or cervical and vaginal pathology, HPV infection, HPV type, histopathological findings, immunosuppression, and type of VaIN-targeted treatment.

### 2.2. Inclusion and Exclusion Criteria

Inclusion criteria were (1) women referred to our tertiary academic center between January 2019 and June 2024, (2) abnormal findings on colposcopic examination of the vaginal wall, and (3) availability of histopathological confirmation following colposcopy-directed vaginal biopsy. Exclusion criteria included (1) incomplete clinical or pathological data, (2) prior diagnosis or treatment for invasive vaginal cancer, and (3) concurrent invasive malignancies at other genital tract sites. During the study period, biopsy samples were taken from 48 women. Twenty-one patients underwent multiple colposcopy-directed biopsies over a follow-up period of 2 to 5 years. The decision to perform repeated biopsies was based on persistent or progressive cytological abnormalities, continued HPV positivity (especially high-risk types), or the presence of suspicious findings on colposcopic follow-up, as per standard clinical protocols at our institution.

### 2.3. Biopsy Protocol and Patient Grouping

The distribution of biopsies was as follows: 33% of patients had two biopsies, 19% had three, 19% had four, 9% had five, 14% had six, and 4% of patients underwent eight biopsies. VaIN was confirmed in 24 patients at the time of the initial biopsy. Additionally, 8 patients showed inflammatory changes, 10 had no signs of dysplasia, 2 exhibited koilocytosis, and 4 presented with other abnormalities. Of the VaIN cases diagnosed throughout the follow-up period, 52% were classified as VaIN 1, 30% as VaIN 2, and 18% as VaIN 3. When considering all patients who underwent biopsy, the prevalence of VaIN 1, 2, and 3 was 29%, 17%, and 10%, respectively. The detection rate of VaIN (50%) among patients undergoing colposcopy-directed biopsy is higher than general population estimates. This likely reflects the referral bias inherent in tertiary academic centers, where patients are referred with pre-existing suspicion for intraepithelial pathology.

### 2.4. Outcome Measures

Primary outcomes included diagnosed lesion regression and progression rates, cytology evolution, recurrence rate, treatment success, and correlation between specific lesion types and risk factors.

### 2.5. Statistical Analysis

The data were entered using Microsoft Office Excel software version 14.0 and analyzed using the Statistical Package for Social Sciences (SPSS) 30.0 (Armonk, NY, USA). Data are expressed as mean ± standard deviation. The analysis of categorical variables was conducted using the Chi-square (χ^2^) tests of independence. The Shapiro–Wilk *W* test was used to check whether the quantitative variable came from a normally distributed population. The Levene (Brown–Forsythe) test was used to test the equality of two variances. The significance of differences between the two groups was tested by the Student *t*-test for normally distributed data or the Mann–Whitney *U* test for non-normally distributed variables. In cases where more than two independent groups were compared, the Kruskal–Wallis test was applied for non-parametric data. A two-tailed *p*-value < 0.05 was considered statistically significant. Where applicable, 95% confidence intervals (CIs) were provided alongside *p*-values to estimate the precision of effect sizes.

## 3. Results

The demographics of the 48 women included in the study are shown in Table 1**.** We then divided our patients into two subgroups: the first included women with a confirmed diagnosis of VaIN (n = 24), and the second included patients without VaIN. This group included patients with coilocytosis, chronic vaginitis, and other benign lesions without dysplasia, as well as patients with no pathological features (n = 24). The first group was further divided into two subgroups based on lesion severity—VaIN1 and VaIN2+ (comprising VaIN2 and VaIN3). We analyzed the associations between different risk factors and the presence of VaIN 1–3 lesions compared to the second group. The results of our comparative analysis are shown in Table 2. HPV infection and a history of cervical pathology were positively associated with the presence of VaIN lesions in the patients analyzed (P = 0.03 and P = 0.05, respectively). A similar analysis was performed between the groups of patients with VaIN1 and VaIN2+ (Table 3). In this case, both HPV infection and HPV HR infection correlated with a higher risk of developing VaIN2+ (P = 0.02 and P = 0.04, respectively). Notably, the youngest patient diagnosed with VaIN2+ was 24 years old, and the only risk factor identified in her case was the presence of ASC-US on cytology. No other statistically significant differences were identified. We also aimed to analyze the association between HPV infection and cytology results in the analyzed cohort (Table 4). Our study corroborates the well-known association between LSIL and HPV infection (P = 0.017), while NILM correlated with cervical HPV negativity (P < 0.001). Other cytology results were not significantly associated with infection. In the HPV-positive group, 13 (52%) had LSIL and seven (28%) had HSIL, compared to only four (17%) and two (9%) in the HPV-negative group, respectively. We also aimed to investigate the relationship between cytology results and histopathological findings from colposcopic biopsies. Of the 32 women with abnormal cytology, colposcopic examination revealed abnormalities in the majority of cases, and only 12 patients (37.5%) had no detectable lesions. Among the 11 patients diagnosed with VaIN2+, the cytology results were variable. Five patients (45.5%) were diagnosed with HSIL and four patients (36.4%) with LSIL. In addition, two patients (9.1% each) had an abnormal cytology result, one ASC-US and one ASC-H. Regarding the treatment of patients with VaIN, 10 (41.7%) underwent laser ablation, three (12.5%) surgical excision, five (20.8%) anti-inflammatory treatment, and seven (29.2%) observation. Treatment depended on the severity of the lesions. Observation was preferred in patients with VaIN1, while surgical treatment was used in patients with VaIN2+. This included excisional treatment with resection of the lesion or laser ablative therapy. In patients who underwent multiple biopsies, disease regression was observed in 11 cases, while four lesions persisted, four progressed, and two recurred. In patients with VaIN2+, lesions were mainly attributed to treatment, with little or no spontaneous regression, whereas VaIN1 lesions tended to regress under observation alone. Among the 11 cases of regression observed, 54.5% of patients were HPV-positive, but only 18.2% had high-risk HPV types (*p* = 0.04), indicating that regression is less likely to occur in the presence of high-risk HPV infections. A history of two or more pregnancies was more common among patients with regression (54.5%) compared to those without regression (40%) (*p* = 0.124). No apparent association between patient age and the occurrence of regression was observed. Notably, 36.4% of patients with regression had a history of immunosuppressive therapy (*p* = 0.087). In the treatment assessment for VaIN, the group only receiving regular follow-up showed a relatively low success rate, with 50% of patients experiencing regression, 25% showing progression, and 25% having persistent changes (*p* = 0.07). In the anti-inflammatory therapy group, 75% of patients experienced regression, with only 25% showing persistent lesions (*p* = 0.03). The laser ablation group showed a 44.4% regression rate, with 33.3% of patients exhibiting progression, and 22.2% experiencing recurrence (*p* = 0.103). These findings likely reflect that laser ablation is typically reserved for more advanced or multifocal stages of VaIN, where lesions are extensive and more challenging to eradicate. As a result, this group tends to exhibit higher rates of disease progression and recurrence compared to patients treated with conservative or topical therapies. Regarding the time to regression and progression based on the severity of VaIN, patients with VaIN 1 lesions required an average of 19.14 months to achieve regression. In contrast, patients with VaIN 2+ lesions had a significantly shorter time to regression, averaging 12.3 months (*p* = 0.03).

## 4. Discussion

**Figure 3:**
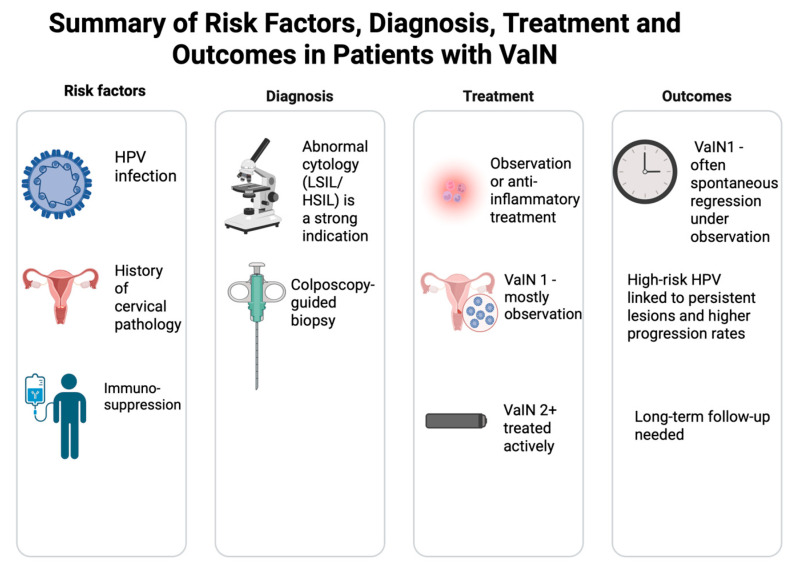
Summary of key results of the study. Created with biorender.com.

Our study confirms the strong association between HPV infection, particularly high-risk subtypes, and the development of high-grade VaIN (VaIN 2+), consistent with the existing literature [7,10,14,23]. Notably, 90.9% of patients with VaIN 2+ were HPV-positive, supporting the critical role of persistent HR HPV in the pathogenesis of advanced intraepithelial lesions. This finding closely mirrors prior studies, including De Vuyst et al., who demonstrated that over 80% of high-grade VaIN cases are attributable to oncogenic HPV types [1]. Our data thus reinforce the established pathogenic role of persistent HR HPV in vaginal intraepithelial neoplasia. A brief summary of key results is shown in Figure 3.

A history of cervical pathology significantly increased the likelihood of developing VaIN in our cohort (P = 0.05), reflecting the known link between cervical and vaginal neoplasia. While prior hysterectomy was not statistically significant, its presence in nearly 30% of VaIN patients echoes findings by Kim et al. and Cao et al., suggesting post-hysterectomy patients remain at risk, particularly for multifocal and apically located lesions [14,20,23]. The correlation between cytology and histology was variable in our cohort. While most high-grade VaIN lesions were preceded by abnormal cytology, a considerable proportion of VaIN 2+ cases had only LSIL or ASC-US findings. This highlights the diagnostic limitations of cytology alone in detecting VaIN, especially in atrophic or post-surgical vaginal mucosa [23,24]. Our findings suggest a conservative approach may be appropriate for VaIN 1, with 11 cases showing spontaneous regression, aligning with other observational studies [10,20]. This discrepancy between cytological and histopathological findings is a well-documented challenge in VaIN diagnosis. Gunderson et al. reported that up to 40% of VaIN lesions are not detected by colposcopy alone, especially in postmenopausal women, where atrophic changes may obscure lesions [7]. Our findings mirror this limitation, as a substantial proportion of high-grade lesions were preceded by only LSIL or ASC-US cytology results. These results emphasize the importance of integrating histological confirmation, iodine staining, and multifocal biopsy even when cytological findings appear mild.

In contrast, most VaIN 2+ cases required ablative or surgical treatment, with limited evidence of spontaneous regression, supporting more active management of high-grade lesions [22,23]. Although only two patients experienced lesion recurrence, and four had persistent disease, these findings underscore the need for long-term surveillance, particularly in HR HPV-positive patients or those treated with less aggressive therapies. This aligns with the recurrence rates reported by Kim et al. in large-scale cohorts [20].

The findings of this retrospective cohort study contribute to a growing body of evidence highlighting the diagnostic, therapeutic, and epidemiological complexities surrounding vaginal intraepithelial neoplasia (VaIN). Although VaIN remains a relatively rare premalignant condition, the detection rate of 50% in women referred for colposcopic evaluation of suspicious vaginal lesions in our study suggests a potentially higher-than-anticipated prevalence, particularly in a tertiary referral population. This aligns with previous estimates that place VaIN incidence between 0.2 and 0.4 per 1000 women, with increasing detection attributed to broader implementation of HPV testing, cytological screening, and improved colposcopic techniques [3,7]. However, the 50% detection rate in our biopsy cohort is notably higher, reflecting the enriched diagnostic yield in a tertiary referral setting. Similarly elevated rates were observed by Gunderson et al., who evaluated women with prior abnormal cytology, and Sillman et al., who reported increased prevalence in high-risk populations [4,7].

A defining feature of our study is the strong correlation between HPV infection and the presence and severity of VaIN lesions. Specifically, HPV positivity was significantly associated with the occurrence of any-grade VaIN (P = 0.03), and high-risk HPV (HR HPV) subtypes were more prevalent among patients with VaIN 2+ (P = 0.04). This is consistent with previous reports underscoring the central role of persistent HR HPV in the pathogenesis of high-grade vaginal lesions [3,20]. Notably, De Vuyst et al. demonstrated in a meta-analysis that over 80% of high-grade VaIN cases are attributable to oncogenic HPV types, primarily HPV 16 and 18, but with growing recognition of other high-risk genotypes such as HPV 45, as also observed in our cohort [1].

Interestingly, despite the established role of HPV, not all VaIN cases in our study were HPV-positive, suggesting a multifactorial etiology that may include immunological, hormonal, and microbiological factors. This notion is supported by Mitra et al., who found that alterations in the vaginal microbiota—particularly a decrease in *Lactobacillus* spp. and increased microbial diversity—are associated with higher-grade cervical and vaginal neoplasias [13]. While we did not assess the vaginal microbiome directly, future studies may benefit from integrating microbiota profiling into VaIN risk stratification. This observation supports the notion of a multifactorial etiology in VaIN pathogenesis. Recent evidence by Mitra et al. and Brusselaers et al. highlights the role of the vaginal microbiome, particularly the depletion of *Lactobacillus* spp. and increased microbial diversity, as a co-factor in high-grade intraepithelial lesions [12,13]. While our study did not assess microbiota composition, future studies may benefit from incorporating vaginal microbiome analysis into risk-stratification models, especially for HPV-negative cases.

The demographic and clinical profiles of affected patients in our study also offer important insights. Although prior cervical pathology was significantly associated with VaIN diagnosis (P = 0.05), other variables traditionally associated with HPV-related disease—including age, parity, smoking, and immunosuppression—did not reach statistical significance. Nevertheless, prior studies have implicated these factors, particularly immunosuppression, as important co-factors in the persistence and progression of HPV-related lesions [10,11]. Our relatively small sample size may have limited the detection of these associations. However, it is important to recognize that over one-quarter of our cohort had undergone a hysterectomy, often for cervical dysplasia, underscoring the need for long-term surveillance in this population. Indeed, post-hysterectomy VaIN is increasingly recognized as a significant clinical entity, often presenting in the vaginal vault and more likely to be multifocal and high-grade [14,15].

A particularly compelling finding in our study was the diagnostic discrepancy between cytological findings and histopathological outcomes. Among patients with abnormal cytology, over 60% had biopsy-confirmed lesions; however, 37.5% showed no visible abnormalities on colposcopy. This supports earlier studies emphasizing the limitations of visual inspection alone. For example, Gunderson et al. noted that up to 40% of VaIN lesions are not visible during routine colposcopy, especially in postmenopausal women with atrophic epithelium and type 3 transformation zones [7]. As such, persistent cytological abnormalities—especially ASC-H and HSIL—should prompt thorough evaluation, including iodine staining and colposcopically directed biopsies of all vaginal quadrants, even in the absence of visible lesions.

Treatment in our study was guided by lesion grade and clinical context. Observation was appropriately selected for low-grade lesions (VaIN 1), consistent with the natural history of spontaneous regression reported in up to 60–80% of cases [25]. In contrast, high-grade lesions (VaIN 2/3) were treated more aggressively, with laser ablation as the predominant method. Our findings align with those of Bogani et al., who showed that laser treatment is an effective and well-tolerated option for high-grade VaIN, with lower recurrence compared to topical agents and favorable tissue preservation [26]. Surgical excision was employed in a minority of cases, primarily when multifocality or suspicious margins raised concern for invasive disease. Notably, excisional treatments offer the advantage of histopathologic margin assessment but carry risks of anatomic distortion and postoperative morbidity, especially in postmenopausal patients [27].

The evidence that treatment, rather than lesion grade, was the main factor influencing regression time adds to a growing body of literature suggesting that clinical outcomes in VaIN can be significantly affected by therapeutic intervention. Among patients undergoing repeat biopsies, four lesions persisted, four progressed, and two recurred. This finding is consistent with recurrence rates reported in the literature, which range from 15 to 30%, depending on treatment method and host factors [28]. Interestingly, recurrence was not confined to patients with known risk factors, as even younger patients without classical risk features exhibited high-grade disease, a phenomenon reported elsewhere [29]. These observations advocate for individualized follow-up protocols based not only on histological grade but also on host immune status, HPV persistence, and prior treatment response. Moreover, the impact of active intervention on regression time in our cohort deserves particular attention.

Importantly, the observed difference in time to regression between VaIN 1 and VaIN 2+ lesions in our cohort warrants closer consideration. While it might initially appear counterintuitive that patients with higher-grade VaIN 2+ exhibited faster regression than those with low-grade lesions, our analysis suggests that this phenomenon is more likely attributable to differences in treatment strategies rather than the inherent biological behavior of the lesions. Indeed, 90.9% of patients with VaIN 2+ underwent active intervention—either laser ablation or surgical excision—compared to only 33.3% of VaIN 1 patients, most of whom were managed conservatively. This observation aligns with findings from other studies that emphasized that active treatment of high-grade VaIN lesions results in higher rates of early histological clearance [7,23]. Furthermore, it underscores the critical role of prompt and appropriate management in achieving lesion regression, especially for lesions associated with high-risk HPV persistence. The evidence that treatment, rather than lesion grade, was the principal determinant of regression time adds to a growing body of literature suggesting that clinical outcomes in VaIN are heavily modifiable by therapeutic intervention [20,24]. These findings advocate for a nuanced approach to VaIN management, recognizing that even low-grade lesions should be carefully monitored and, in selected cases, treated proactively to prevent persistence or progression, particularly in patients with concomitant risk factors such as persistent HPV infection or immunosuppression [1,14]. Future prospective studies are needed to further delineate the interplay between lesion severity, treatment method, and regression kinetics, potentially leading to more individualized management strategies.

Our results also support the growing emphasis on HPV vaccination as a preventive strategy. Prophylactic HPV vaccination, particularly with the quadrivalent and nonavalent vaccines, has demonstrated efficacy against VaIN 1 and promising results for VaIN 2/3 prevention [30]. The Dillner et al. randomized trial reported complete protection against low-grade VaIN lesions in vaccinated women and up to 72–100% efficacy against high-grade lesions [30]. Integration of catch-up vaccination for women with a history of CIN or VaIN could potentially mitigate recurrence risk, particularly when combined with regular surveillance. In addition to vaccination, expanding HPV screening—particularly with genotyping—could offer a cost-effective strategy for early detection of VaIN and prevention of progression. Future studies should assess the economic and clinical utility of incorporating HPV screening into routine post-CIN and post-hysterectomy surveillance protocols.

Finally, although not within the scope of this study, considering quality-of-life outcomes is becoming increasingly critical in evaluating VaIN management. Excisional therapies, while curative, may result in scarring, dyspareunia, or anatomical narrowing, especially in older women. Topical agents such as imiquimod, although less invasive, are associated with local irritation and variable efficacy [22]. In this regard, the consensus guidelines from the European Society of Gynaecological Oncology (ESGO) advocate for a patient-centered approach that balances oncologic safety with functional and psychosexual outcomes [23].

## 5. Conclusions

The retrospective analysis presented here confirms that VaIN, though uncommon, requires heightened clinical suspicion and multimodal diagnostic strategies to ensure accurate detection and appropriate management. HPV infection, particularly with high-risk genotypes, remains the most significant driver of disease onset and progression, and HPV genotyping should be incorporated into routine evaluation. While low-grade lesions may regress under observation, high-grade VaIN necessitates definitive treatment—preferably laser ablation or excision—followed by long-term follow-up due to the risk of recurrence. The findings support the existing literature and reinforce the urgent need for standardized guidelines and preventive strategies, including vaccination, to reduce the burden of this challenging and often underrecognized disease.

## Figures and Tables

**Table 1:** Characteristics of patients included in the analysis.

Demographic Categories	
Age (mean)	52.9 ± 13.3
Number of past pregnancies (mean)	1.1 ± 1.0
History of cervical pathology	14 (29.2%)
Prior hysterectomy due to cervical pathology	12 (25.0%)
HPV infection	25 (52.1%)
HR HPV	17 (35.4%)
VaIN1	13 (27.1%)
VaIN2+	11 (22.9%)
No evidence of dysplasia	20 (42%)
Others (i.e., atrophic vaginitis, inclusion cysts, squamous metaplasia, or non-specific/reactive epithelial changes)	4 (8.3%)

**Table 2:** Risk factor presence in the VaIN and non-VaIN groups.

Risk Factor	VaIN (n = 24)	Non-VaIN (n = 24)	*p* Value
Smoking	2 (8.3%)	2 (8.3%)	0.185
HPV infection	17 (70.8%)	8 (33.3%)	0.03
HR HPV	9 (37.5%)	8 (33.3%)	0.760
Immunosuppression	7 (29.2%)	5 (20.8%)	0.487
History of cervical pathology	10 (41.7%)	4 (16.7%)	0.05
Prior hysterectomy	7 (29.2%)	5 (20.8%)	0.626
Age (mean ± SD)	50.8 ± 13.1	51.6 ± 13.9	0.269
Number of pregnancies (mean ± SD)	1.3 ± 1.2	1.3 ± 1.1	0.723

**Table 3:** Risk factor presence in the VaIN 1 and VaIN2+ groups.

Risk Factor	VaIN 1 (n = 13)	VaIN 2+ (n = 11)	*p* Value
Smoking	0 (0%)	2 (18.2%)	0.189
HPV infection	7 (53.8%)	10 (90.9%)	0.02
HR HPV	2 (22.2%)	7 (63.6%)	0.04
Immunosuppression	2 (22.2%)	5 (45.5%)	0.291
History cervical pathology	3 (33.3%)	3 (27.3%)	0.448
Prior hysterectomy	3 (23.1%)	4 (36.3%)	0.668
Age (mean ± SD)	49.8 ± 12.7	51.6 ± 13.9	0.178
Number of pregnancies (mean ± SD)	1.5 ± 1.3	1.3 ± 1.1	0.871

**Table 4:** Correlation of cervical human papillomavirus (HPV) result and liquid-based cytology. Values are presented as absolute numbers and percentages.

	**Positive Baseline Cervical HPV (*n* = 25)**	**Negative Baseline Cervical HPV (*n* = 23)**	
Cervical cytology at first control	*n* (%)	*n* (%)	*p* value
NILM	2 (8%)	12 (52%)	<0.001
LSIL	13 (52%)	4 (17%)	0.017
HSIL	7 (28%)	2 (9%)	0.890
ASC-US	1 (4%)	5 (22%)	0.770
ASC-H	2 (8%)	0 (0%)	0.490

## Data Availability

The datasets used and/or analyzed during the current study are available from the corresponding author upon reasonable request.
